# Perspectives of Stakeholders About an Early Result Acceptance Program to Complement the Residency Match in Obstetrics and Gynecology

**DOI:** 10.1001/jamanetworkopen.2021.24158

**Published:** 2021-10-11

**Authors:** Abigail Ford Winkel, Helen K. Morgan, Oluwabukola Akingbola, Keli Santos-Parker, Erin Nelson, Erika Banks, Nadine T. Katz, Jessica L. Bienstock, David Marzano, Maya M. Hammoud

**Affiliations:** 1 New York University Grossman School of Medicine, New York; 2 University of Michigan Medical School, Ann Arbor; 3 University of Minnesota Medical School, Minneapolis; 4 University of Michigan, Ann Arbor; 5 Joe R. and Teresa Lozano Long School of Medicine at UT Health San Antonio, San Antonio, Texas; 6 Albert Einstein/Montefiore Medical Center, Bronx, New York; 7 Johns Hopkins University School of Medicine, Baltimore, Maryland

## Abstract

**Importance:**

The residency application process is flawed, costly, and distracts from the preparation for residency. Disruptive change is needed to improve the inefficiencies in current selection processes.

**Objective:**

To determine interest in an early result acceptance program (ERAP) among stakeholders in obstetrics and gynecology (OBGYN), and to estimate its outcome in future application cycles.

**Design, Setting, and Participants:**

Surveys of stakeholders in March 2021 queried interest in ERAP across the US. Respondents included OBGYN residency applicants, members of the Association of American Medical Colleges Group on Student Affairs, OBGYN clerkship directors, and residency program directors. Statistical analysis was performed from March to April 2021.

**Exposures:**

Respondents completed surveys sent by email from the Association of American Medical Colleges (to OBGYN applicants and members of the Group on Student Affairs), the Association of Professors of Gynecology and Obstetrics (to clerkship directors), and the Council on Resident Education in Obstetrics and Gynecology (to program directors).

**Main Outcomes and Measures:**

Applicants and program directors indicated their interest in participating in ERAP, and clerkship directors and members of the Group on Student Affairs indicated their likelihood of recommending ERAP using a 5-point Likert scale.

**Results:**

Respondents included 879 (34.0%) of 2579 applicants to OBGYN, 143 (50.3%) of 284 residency program directors, 94 (41.8%) of 225 clerkship directors, and 51 (32.9%) of 155 student affairs deans. The majority of respondents reported being either somewhat or extremely likely to participate in ERAP, including 622 applicants (70.7%) and 87 program directors (60.8%). Interest in ERAP was independent of an applicant’s reported board scores, medical school type, race, number of applications submitted, or number of interviews completed. Among program directors, those at university programs were more likely to participate. Stakeholders supported a limit of 3 applications for ERAP, to fill 25% to 50% of residency positions. Estimating the outcome of ERAP using these data suggests 26 280 to 52 560 fewer applications could be submitted in the regular match cycle.

**Conclusions and Relevance:**

Stakeholders in the OBGYN application process expressed broad support for the concept of ERAP. The majority of applicants and programs indicated that they would participate, with potentially substantial positive impact on the application process. Careful pilot testing and research regarding implementation are essential to avoid worsening an already dysfunctional application process.

## Key Points

**Question:**

Are key stakeholders in obstetrics and gynecology interested in an early result acceptance program, and what are possible outcomes of such a program?

**Findings:**

This survey study of 879 applicants, 143 program directors, 94 clerkship directors, and 51 student affairs deans revealed broad support for an early result acceptance program, with respondents indicating support for allowing 3 early applications and offering 25% to 50% of first-year resident positions. Based on survey responses, an early result acceptance program could reduce the number of applications in the regular cycle by 26 280 to 52 560 applications.

**Meaning:**

The findings of this study suggest broad support for an early result acceptance program in obstetrics and gynecology, which could provide the opportunity to improve the application experience for applicants and program directors.

## Teaser

This survey study examines perspectives among stakeholders in obstetrics and gynecology regarding the potential for an early decision process to complement the current residency match process.

## Introduction

The residency application process has become increasingly problematic, with heavy burdens facing applicants and residency programs.^1^ Applicants exceed the available positions in graduate medical education (GME) and apply to more programs each year.^1,2^ Regardless of their competitiveness, applicants adopt an “over-apply, over-interview” strategy.^3^ The costs of these behaviors are financial, educational, and emotional. Efforts to match into residency displace educational efforts during the last year of medical school, which could ensure an effective transition from student to resident.^4^ Increasing numbers of applications strains programs and forces them to rely on numerical metrics rather than performing holistic reviews. According to the 2020 Program Director Survey conducted by the National Resident Matching Program (NRMP), 45% of applications are rejected based on screening metrics alone.^5^ For students who identify their top programs before submitting applications—a program at their own medical school, where they did a visiting elective, or where they want to live—little is gained through excessive applications and interviews. Geography itself is a key determinant in applicants’ final ranking, a decision likely made independent of the application process.^6^ Some suggest that students matching into their medical school’s own residency programs could improve their training and the care of their future patients through an enhanced transition to residency.^7^ If some students matched early into their top programs, this could leave other applicants and programs the opportunity to adopt a more thoughtful approach for the regular application cycle.

The Coalition for Physician Accountability proposed recommendations to improve the transition from undergraduate medical education (UME) to GME, citing application inflation as a “root cause of the current dysfunction in the UME-GME transition.”^8^ Early applications to undergraduate colleges is a well-established part of college admissions, widely embraced by students and colleges.^9^ Residency applications for neurology, neurosurgery, ophthalmology, and urology occur prior to the NRMP main residency match.^10^ An optional early result acceptance program (ERAP) has been proposed to allow for a 2-stage match with a limited group of applicants matching prior to the regular application cycle.^11^ A qualitative study of stakeholders in the match process proposed a summer match as a means of reducing cost and other negative consequences of application inflation.^12^

The aim of this study is to explore the opinions of stakeholders in obstetrics and gynecology to determine their interest in participation in an ERAP, determine ideal parameters for a proposed ERAP in terms of application numbers and available positions, and use these numbers to estimate the potential impact of ERAP on the application process.

## Methods

Stakeholders in the OBGYN application process were queried using an electronic survey about their interest in an ERAP as part of the Right Resident, Right Program, Ready Day One (RRR) initiative led by the Association of Professors in Gynecology and Obstetrics (APGO) and funded by the American Medical Association Reimagining Residency initiative. Informed consent was not included in the surveys as the University of Michigan institutional review board determined that the study was exempt from continuing review because it carried no more than minimal risk to participants. Surveys queried stakeholder demographics and application and interview experiences (eAppendix in the Supplement). Members of the RRR team designing the survey included OBGYN educators in UME and GME, administrative leaders, medical students and residents attending allopathic and osteopathic schools, as well as international medical graduate applicants. Content validity of the survey was established through literature review and consultation with experts. Response process validity was explored through piloting the survey with representatives of stakeholder groups.

Surveys were distributed electronically to medical students and members of the Group on Student Affairs (GSA) by the Association of American Medical Colleges, to medical school clerkship directors (CDs) by APGO, and to residency program directors (PDs) by the Council on Resident Education in Obstetrics and Gynecology. Surveys were distributed in March 2021 after the submission deadlines for applicant and program match rank lists, and prior to the results of the 2021 NRMP match being released to ensure rank lists and match results did not influence the answers to the survey. Applicants provided demographic information including self-reported race and ethnicity on the survey using predefined options and free-text options. Gender and age demographic information were not collected. The overall response rate was 36.0% (1167 of 3243). This study followed the American Association for Public Opinion Research (AAPOR) reporting guideline.^13^

In the survey, ERAP was described as a binding early decision process where students would apply to a limited number of programs. Programs could make a portion of first-year positions available through ERAP, with results announced in September prior to the opening of the main match. Respondents were asked to indicate their likelihood of participating in ERAP (students and PDs) or recommending ERAP to students (CDs and GSA members) using a 5-point Likert scale. Respondents suggested the number of applications and available residency positions they believed should be included.

### Statistical Analysis

Descriptive statistics explored the demographics of each stakeholder group and their interest in ERAP. Applicant characteristics for survey respondents were compared with the overall population of applicants in OBGYN in 2020^5^ using χ^2^ tests. The association between PD and applicant characteristics and interest in participating in ERAP were assessed using χ^2^ tests and Kruskal-Wallis tests for categorical data, and 1-way analysis of variance tests for interval data (numbers of applications and interviews). Using the proposed numbers of applications and percentage of positions recommended by the stakeholder groups, we estimated the outcome of an ERAP on the number of applications in the regular match. Two reviewers (E.N. and H.K.M.) analyzed free-text comments by examining comments, identifying recurring themes, and organizing them into categories. Serial review of comments, discussions among the authorship team, examination of trends and quantitative data findings helped to delineate opportunities and challenges presented by ERAP.

Statistical analysis was performed using SPSS Statistics version 25 (IBM Corp) from March to April 2021. The threshold for statistical significance was *P* < .05.

## Results

Respondents to the survey included 879 (34.0%) of 2579 applicants to OBGYN residency, 143 (50.3%) of 284 OBGYN residency PDs, 94 (41.8%) of 225 CDs, and 51 (32.9%) of 155 student affairs deans after removing incomplete responses. Applicant respondents included 564 (66%) from US allopathic medical schools, 149 (17%) from US osteopathic schools, and 145 (16%) from international schools. Most undergraduate medical education faculty came from MD programs, including 91 clerkship directors (97%) and 51 deans (100%). Among PDs, 76 (53%) were at university programs, 43 (30%) were at community-based/university affiliated programs, 22 (15%) were at community programs and 1 (1%) was at a military program. Among applicants, 489 (56%) were White. Demographics of the groups are outlined in Table 1. Compared with the overall applicant pool, the survey respondents had similar distribution between MD, DO, and international medical graduate applicants, and had similar United States Medical Licensing Examination (USMLE) or Comprehensive Osteopathic Medical Licensing Examination (COMLEX) scores (eAppendix in the Supplement). Most respondents to all surveys reported being either somewhat or extremely likely to participate or recommend participation in ERAP, including 622 applicants (70.7%), 87 PDs (60.8%), 70 clerkship directors (74.7%) and 34 student affairs deans (66.7%) (Table 2). There were 72 applicants (8.2%) and 15 PDs (10.4%) who stated that they would be “extremely unlikely” to participate in ERAP.

**Table 1.** Demographic Information Reported by Survey Respondents

Characteristic	Respondents, No. (%)			
	Applicants (n = 879)	Clerkship directors (n = 94)	Student affairs deans (n = 51)	Residency program directors (n = 143)
Degree pursued or degrees offered				
MD (US)	564 (66)	91 (97)	51 (100)	NA
DO	149 (17)	3 (3)	0	NA
MD (IMG)	145 (16)	0	0	NA
Residency program type				
University	NA	NA	NA	76 (53)
Community-based/university affiliated	NA	NA	NA	43 (30)
Community-based	NA	NA	NA	22 (15)
Military	NA	NA	NA	1 (1)
Board exam scores				
USMLE<200 or COMLEX<487	41 (5)	NA	NA	NA
USMLE 200-220 or COMLEX 488-575	223 (25)	NA	NA	NA
USMLE 221-240 or COMLEX 576-660	366 (42)	NA	NA	NA
USMLE 241-260 or COMLEX 661-742	207 (24)	NA	NA	NA
USMLE>260 or COMLEX>742	19 (2)	NA	NA	NA
Race and ethnicity^a^				
American Indian or Alaska Native	7 (1)	NA	NA	NA
Asian	126 (14)	NA	NA	NA
Black or African American	86 (10)	NA	NA	NA
Hispanic, Latino, or of Spanish origin	85 (10)	NA	NA	NA
Native Hawaiian or other Pacific Islander	2 (<1)	NA	NA	NA
White	489 (56)	NA	NA	NA
Other^b^	84 (9)	NA	NA	NA

Abbreviations: COMLEX, Comprehensive Osteopathic Medical Licensing Examination; IMG, international medical graduate; NA, not available; USMLE, United States Medical Licensing Examination.

^a^ Indicates primary identification selected. Participants were given the option to select other race and ethnicity identifications.

^b^ Other was a choice that the respondents could select, and they could then write free-text responses. Usually those selecting this race and ethnicity category would indicate that they identified with several different categories included above.

**Table 2.** Stakeholder Interest in ERAP

Response	No. (%)			
	Would you participate in ERAP?		Would you recommend students participate in ERAP?	
	Applicants (n = 853)	Residency directors (n = 143)	Clerkship directors (n = 93)	Graduate student affairs deans (n = 51)
Extremely likely	336 (39)	32 (22)	43 (46)	18 (35)
Somewhat likely	286 (33)	55 (39)	27 (29)	16 (31)
Neither likely or unlikely	76 (9)	29 (20)	9 (10)	8 (16)
Somewhat unlikely	83 (10)	12 (8)	10 (11)	2 (4)
Extremely unlikely	72 (8)	15 (10)	4 (4)	7 (14)

Abbreviation: ERAP, early result acceptance program.

For applicants, there was no association between an applicant’s likelihood of participating in ERAP and their USMLE or COMLEX scores (*H*[4] = 5.2; *P* = .28), identifying as White compared with applicants identifying as other races and ethnicities (χ^2^ = 6.3; *P* = .16), number of applications submitted (*F*_161,684_ = 1.16, *P* = .11) or number of interviews received (F_39,806_ = 1.04; *P* = .40). Students from US allopathic, osteopathic, and international medical schools expressed similar likelihood of participation in ERAP (χ^2^ = 13.4; *P* = .10). Among 59 applicants participating in the match as a couple, 34 (57.6%) were somewhat or extremely likely to participate in ERAP, compared with 588 (71.7%) of the 820 applicants not participating in the match as a couple (χ^2^ = 15.5; *P* = .05).

For PDs, interest in ERAP was not associated with program size, (*F*_4,136_ = 1.13; *P* = .35), number of applications received (*F*_4,137_ = 2.07; *P* = .09), or interviews offered (*F*_4,136_ = 0.56; *P* = .70). Among PDs, a greater proportion of university PDs (51 [67.1%]) were somewhat or extremely likely to participate in ERAP compared with PDs at community programs (9 [40.9%]) and PDs at community-based, university-affiliated programs (25 [59.5%]) (χ^2^ = 84.1; *P* < .001).

### Parameters for ERAP

Regarding the number of applications that should be submitted in ERAP, applicants favored a mean (SD) of 3.7 (1.2) applications, clerkship directors favored 3.1 (1.1) applications and student affairs deans favored 3.8 (1.4) applications. PDs provided free-text responses to the number of positions they felt should be offered in ERAP, and these ranged from 25% to 100% of slots (median [IQR], 25% [25%-50%], mean 39.0% [17.4%]).

### Estimated Outcomes of ERAP on Match

Applicants in our survey submitted a mean (SD) of 72.4 (40.6) applications and a median (IQR) of 61 (18-104) applications; and applicants received a mean (SD) of 11.8 (8.5) interviews and a median (IQR) of 11 (1-21) interviews. When asked about the top 3 programs they had identified before the application process, 673 applicants (76.6%) reported getting an interview with at least 1 of these programs, and 124 applicants (14%) reported getting interviews at all of their top 3 programs. Among applicants who received at least 1 interview at their original top 3 programs, 618 (91.8%) ranked at least 1 of these programs in their final list. Among applicants receiving interviews at their top 3 programs, there were 1155 interviews and 1014 positions later ranked in the top 3, suggesting that 87.8% of these interviews confirmed initial interest in applicants’ top choices for residency.

Using the respondents of this survey as a guide, we modeled how an ERAP would impact the available interview positions in the regular application cycle, depicted in the Figure. In OBGYN, 279 programs offered 1460 positions in the match.^14^ If stakeholders participated as indicated in this survey, we estimated outcomes with ERAP in a future cycle. Among 1873 applicants, 1311 (70%) would participate in ERAP, and 996 (76%) would receive at least 1 interview in their top 3 programs. If 60% of 279 residency programs participated, offering 25% to 50% of first-year resident positions, 365 to 730 positions would be available through ERAP. Because 91% of applicants ranked top programs where they interviewed on their final list, with 1430 applicants, we assumed all positions in ERAP would fill. This would remove 365 to 730 applicants from the regular cycle. With applicants submitting a mean of approximately 72 applications, this could result in 26 280 to 52 560 fewer applications overall, or a mean of 92 to 188 fewer applications to each program. Those applicants receiving interviews at all 3 of their top choice residency programs, applied to a mean (SD) of 59 (17) programs. Estimates of the impact of ERAP on the competitiveness of the application cycle for applicants as well as the administrative burden on programs are presented in Table 3. We modeled the ratio of positions to applicant in the ERAP vs the regular match. The likelihood of matching in ERAP differs depending on the number of positions offered by programs, with ERAP having fewer positions per applicant in the ERAP cycle. The ratio of positions to applicant is projected to be similar following ERAP in the regular cycle (0.71-0.75) to the current ratio of positions to applicant (0.78).

**Figure.**
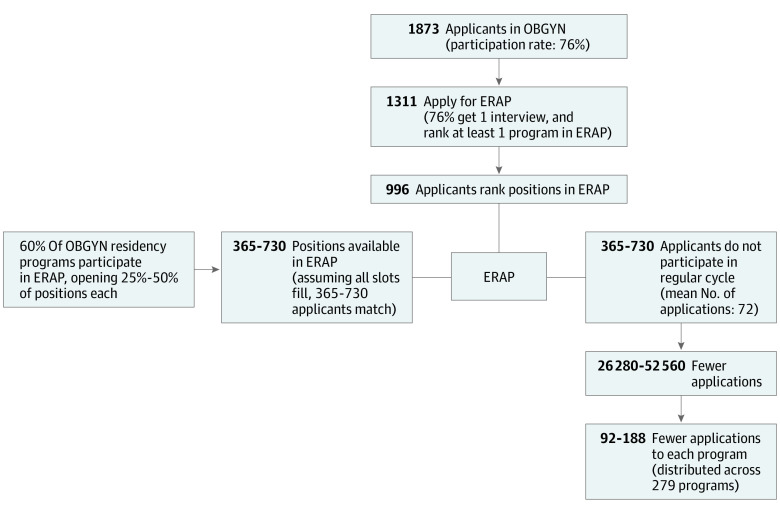
Potential Outcomes of Early Result Acceptance Program in Obstetrics in Gynecology on Regular Application Cycle The figure shows the estimated outcomes of ERAP on the OBGYN match, using responses from stakeholder surveys to estimate future behaviors of applicants and programs. Based on these data, the majority of applicants and programs participate, filling all available positions in ERAP. If 365 to 730 applicants are removed from the regular application cycle, and each applicant submits 72 applications, that could mean 26 280 to 52 560 fewer applications submitted with hundreds of fewer applications required for review at each program. ERAP indicates early result acceptance program; OBGYN, obstetrics and gynecology.

**Table 3.** Estimated Outcomes of an Early Result Application Program on Competitiveness of the Regular Match and Application Numbers

Variable	Current application state	ERAP (with 60% residency programs participating)	
		25% of positions available in ERAP	50% of positions available in ERAP
No. of positions available in ERAP	0	219	438
No. of applicants participating in ERAP^a^	0	1311	1311
No. of applicants participating in the regular match^b^	1873	1654	1435
No. of OBGYN PGY-1 positions in available regular match	1460	1241	1022
Positions: applicant ratio			
In ERAP	NA	0.22	0.33
In regular match	0.78	0.75	0.71
Applications submitted			
In ERAP^c^	0	3933	3933
In regular cycle^d^	134 856	119 088	103 320
Interviews conducted^e^	17 520	15 549	13 578
Applications saved	0	15 768	31 536
Interviews saved	0	1971	3942
Reduction in interviews, %	0	11	23

Abbreviations: ERAP, early result acceptance program; NA, not applicable; OBGYN, obstetrics and gynecology; PGY-1, postgraduate year 1.

^a^ Based on estimates that 70% of applicants would participate in ERAP.

^b^ Based on the assumption that all positions available in ERAP will fill.

^c^ Based on ERAP applicants submitting 3 applications.

^d^ Based on applicants submitting an average of 72 applications.

^e^ Based on programs conducting 12 interviews for each position to fill.

Qualitative analysis of survey respondents revealed positive sentiments regarding the need for organized action within the specialty and substantial change. As one PD stated, “the whole process needs an overhaul.” Stakeholders’ concerns categorized into 3 themes centering on the challenges of implementation. These were (1) fears of introducing additional administrative burden onto programs, (2) increasing applicants’ anxiety, or (3) worsening inequities in the application process. Based on the comments shared and the quantitative analyses performed, the barriers and opportunities presented with ERAP are outlined in Table 4.

**Table 4.** Opportunities and Challenges Presented by an Early Result Acceptance Program

Opportunities	Challenges
Decrease in numbers of applications	Limited applications that may not include applicants’ student subinternship or elective experiences
Decreased costs for applicants and programs	Possible premature closure and lack of discovery of a program that was not originally identified as a top choice
Opportunity for a more holistic review	Ensuring there is adequate residency advising to prepare students for an early match
Applicants’ ability to signal program preferences	Fewer opportunities to obtain letters of recommendation for residency applications
Increased time in final year of medical school to learn and prepare for residency	Additional administrative burden on residency programs during summer months
Increased lead time for personal and family transition to a new city	Concerns regarding stigma associated with “deferral” in the early match
Potential Increased diversity of applicants to rural/smaller programs	Ensuring that anxiety and inequities in the process for students are not increased

## Discussion

Stakeholders in the OBGYN residency application process expressed broad support for an ERAP in this survey study. This study suggests that the majority of respondents would participate, and the majority of them would be successful. Applicants and PDs: those most directly affected by the process, displayed the most pronounced enthusiasm for ERAP. The proposed ERAP should include a limit of 3 applications per applicant, and allow programs to make 25% to 50% of positions available in the first stage of the match. The estimated impact of an ERAP based on these survey data would be a significant decrease in applications in the main match, decreasing costs and anxiety and allowing these applicants to focus on their education. In addition, hundreds of unnecessary applications by competitive applicants would be removed from the pool, facilitating holistic review of applications in the main match. In addition, applying through ERAP would serve as a signaling mechanism to their programs of interest, even for students who did not match through ERAP.

In this study, 76% of applicants received interviews at one of their top 3 program choices, which may translate into the proportion receiving interviews through ERAP. It is reassuring that such a large portion of applicants identify programs where they are viable candidates for a successful match. Applicants’ interest in ERAP was not associated with medical school background, race, board scores or interviews received. Fewer positions per applicant would be available in ERAP than the regular match cycle, and the number of positions per applicant in the main match would be similar after ERAP to the current state. These estimates should alleviate concerns that applicants perceive that participating in ERAP would be necessary for a desirable match. However, matching highly competitive applicants and those with strong preferences through ERAP could result in similar or improved match outcomes for the remaining students. Students in participating in couples match were less likely to participate in ERAP. In this study, university programs were more likely to participate in ERAP than community-based programs. However, it’s possible those programs could benefit if ERAP decreased unnecessary applications to their programs. Early concerns about the practicality of a staged match have been allayed by successful implementation of virtual interviews and a compressed application timeline, changes necessitated in the 2020 to 2021 cycle due to the COVID-19 pandemic.

The cost to applicants could be significantly reduced through ERAP. Residency applicants spend an estimated $340 to $450 per interview.^15^^,^^16^ Estimating $400 per interview in OBGYN, each applicant matching in ERAP who completes 3 interviews instead of the average 12 would save $3600. If between 365 to 730 applicants match using ERAP, this would save $1 314 000 to $2 628 000 overall. Although ERAP prolongs the application cycle, the overall reduction in applications and interviews would significantly reduce administrative burden for programs. Residency program costs for interviews may be as high as $190/applicant in terms of faculty time, not to mention expenditures on materials and receptions.^17^ Across all programs, the estimated savings for 6570 interviews—the number of interviews saved with all programs participating and 50% of positions filling in ERAP—is $1 282 500.

The parameters for ERAP supported by respondents to this survey include a limited ERAP focused on those applicants with strong early preferences and high likelihood of matching in those programs. Careful study must be undertaken to ensure there are no undesired effects of ERAP on underrepresented groups and certain programs. Some fear that applicants who did not match in ERAP would have heightened anxiety and increase the number of applications submitted to the regular cycle. We argue an unsuccessful ERAP application might prompt an applicant to focus their applications on programs they are more likely to match. Our respondents reported submitted an average of 72 applications. There may be a ceiling to the number of programs applicants will apply to as students applying to the most competitive specialties such as dermatology or orthopedic surgery already submit 60 to 80 applications. In weighing the advantages and disadvantages of taking steps toward change, the status quo may be the worst-case scenario.

Other interventions to improve application inflation have been suggested, such as applicants signaling their preference to a limited number of programs. This approach offers promise, but would not reduce the number of applications.^18^ Capping applications disadvantages applicants by forcing them to limit their choices. Although ERAP effectively introduces a cap in the first stage, it is optional and allows all applicants the opportunity of unrestricted applications in the second stage if desired while sending a signal in the first round.

### Strengths and Limitations

This study had strengths and limitations. The strengths of this study are its sampling across stakeholders in the OBGYN application process. Despite a lower response rate, the demographics of the respondents are similar to the NRMP data, and the distribution of applicants’ medical school status and USMLE or COMLEX scores mimics the breakdown of applicants applying nationally. The population of institutional stakeholders is less representative, with more MD-granting programs represented among GSA and CD respondents, and more university PDs responding. We compared only applicants identifying as White to all other races, because applicants could select several racial and ethnic identities, limiting the ability to explore associations that are more specific between racial/ethnic identity and ERAP interest. A pilot of ERAP is necessary to understand the true response to the opportunity among stakeholders. Pilot testing should include assessment of outcomes for residents who match via ERAP across the years of their residency training to measure the quality of the match beyond match rates. ERAP implementation must include careful monitoring of outcomes in order to make necessary changes and mitigate undesired effects on administrative burden, anxiety or inequities for underrepresented groups.

## Conclusions

The current application process drains resources from applicants and programs. A binding early match could reduce congestion within the application cycle, reduce waste, and allow programs and applicants to refocus their efforts during the final year of medical school. Insights gained from an ERAP pilot in OBGYN could inform efforts in other specialties and programs that include specialized tracks, match for postgraduate year 2 positions and other scenarios. Piloting an ERAP with an iterative, continuous quality improvement practice in a single specialty could be very informative to suggesting changes to the current system which would improve the application and match process for all stakeholders.

## Notes

Supplement. **eAppendix.** Supplemental Materials
