# Supplementary material for: Perspectives of Stakeholders About an Early Result Acceptance Program to Complement the Residency Match in Obstetrics and Gynecology
Source: JAMA Netw Open. 2021 Oct 11;4(10):e2124158. doi: 10.1001/jamanetworkopen.2021.24158 (PMC8506230; doi:10.1001/jamanetworkopen.2021.24158)
Supplement: Supplement. — eAppendix. Supplemental Materials [file jamanetwopen-e2124158-s001.pdf]

Supplemental Online Content

Winkel AF, Morgan HK, Akingbola O, et al. Perspectives of stakeholders about an early result acceptance program to complement the residency match in obstetrics and gynecology. *JAMA Netw Open.* 2021;4(10):e2124158. doi:10.1001/jamanetworkopen.2021.24158

eAppendix. Supplemental Materials

This supplemental material has been provided by the authors to give readers additional information about their work.

© 2021 Winkel AF et al. *JAMA Network Open.*

eAppendix. Supplemental Materials

Demographics of survey respondents (2021) compared to National Resident Matching Program (NRMP) data[a]

	**Survey Respondents N(%)**	**NRMP N(%)**	**Chi Squared**	**P Value**
USMLE Step 1 score or COMLEX 1 score				
USMLE<200 or COMLEX <487	41 (5)	50 (3)		
USMLE 200-220 or COMLEX 488-575	223 (26)	418 (26)		
USMLE 221-240 or COMLEX 576-660	366 (43)	746 (46)	4.61	0.33
USMLE 241-260 or COMLEX 661-742	207 (24)	371 (23)		
USMLE >260 or COMLEX >742	19 (2)	22 (1)		
Medical School Status at time of Application				
US Senior MD	548 (64)	1260 (70)		
US Alum MD	16 (2)			
US Senior DO	142 (17)	308 (17)		
US Alum DO	7 (1)		2.8	0.42
IMG US	64 (7)	131 (7)		
IMG US Alum	12 (1)			
IMG Non US	62 (7)	110 (6)		
IMG Non US Alum	7 (1)			

[a]Results from NRMP Data (*Results and Data 2020 Main Residency Match®*, 2020)

© 2021 Winkel AF et al. *JAMA Network Open.*

RRR OBGYN Student Survey 2021

Preview link:

https://umich.ca1.qualtrics.com/jfe/preview/SV_3jIb2XKy9dXHIuq?Q_CHL=preview&Q_SurveyVersionID=current

Start of Block: SURVEY INSTRUCTIONS

Q1 The following questions pertain to the residency application and interview processes in obstetrics and gynecology (OBGYN). Please try to answer all questions to the best of your ability as these are intended to evaluate the changes recommended during the 2020-2021 application cycle. Thank you, we value your input.

Q2 Which of the following Specialty-Wide Standards for the OBGYN Residency Application and Interview Processes are you aware of? CHECK ALL THAT APPLY

- ☐ November 1, 2020 as the deadline for final OBGYN application submission (1)
- ☐ December 23, 2020 as the final applicant status notification by programs (i.e. waitlist, rejection) (2)
- ☐ November 10 and November 17, 2020 for initial release of interview offers, followed by rolling offers (3)
- ☐ 48 hours minimum after an interview invitation email has been sent for the applicant to respond (4)
- ☐ Limiting program interview invitations to the number of interview slots available (5)
- ☐ I am not aware of any of the above standards (6)

© 2021 Winkel AF et al. *JAMA Network Open.*

Q3 Rate the importance of each of the following as it pertains to the OBGYN application and interview processes

	Not at all important (1)	Slightly important (2)	Moderately important (3)	Very important (4)	Extremely important (5)
The same application submission deadline for all programs (1)	○	○	○	○	○
A common date for final application status notification to the applicant (i.e. waitlist, rejection) (2)	○	○	○	○	○
Common pre-determined interview offer date(s) for all programs (3)	○	○	○	○	○
A 48-hour period for applicants to respond to an interview offer (4)	○	○	○	○	○
Limiting the number of interview offers to slots available (5)	○	○	○	○	○

© 2021 Winkel AF et al. *JAMA Network Open.*

Q4 Please fill in the following. Please enter whole numbers- enter 0 if none. If you cannot recall exactly use your best estimate.

◯ Number of applications you submitted (9) ____

◯ Number of interview invitations you received (1) ____

◯ Number of interviews you completed (7) ____

◯ Number of interview invitations you received on November 10 or November 17 (2) ____

◯ Number of interview invitations which indicated you had 48 hours to respond to the interview offer (3) ____

◯ Number of programs that offered you an interview (that was not specified as a wait list) and did not have an interview spot (4) ____

◯ How many specialties did you apply to in addition to OBGYN (enter 0 if none) (8) ____

Display This Question:

If If Please fill in the following. Please enter whole numbers- enter 0 if none. If you cannot recall e... How many specialties did you apply to in addition to OBGYN (enter 0 if none) Is Not Equal to 0

Q5 What was your first specialty choice?

▼ OBGYN (1) ... Other (21)

Display This Question:

If Q5 = Other

Q6 Please enter the name of your first choice specialty

____

© 2021 Winkel AF et al. *JAMA Network Open.*

Q7 Please rank the following interview options which you believe programs should consider in the future from most desirable (1) to least desirable (4). Please assume that in person options have no restrictions (as in pre-COVID times).

_______ All interviews are conducted exclusively virtually (1)
_______ All interviews are conducted virtually with open house in person visit option (2)
_______ All interviews are conducted in person (3)
_______ Applicants have the option of virtual or in person interview (4)

Q8 Thinking about the **top 3 programs you were considering when you applied at the beginning** of the application season, please answer the following questions.

Q9 How many of those initial top programs offered you an interview?

- ◯ 0 (1)
- ◯ 1 (2)
- ◯ 2 (3)
- ◯ 3 (4)

Display This Question:

If Q9 = 0

© 2021 Winkel AF et al. *JAMA Network Open.*

Q10 Why do you believe none of your top choice programs offered you an interview? Please check all that apply.

- ☐ Demographic data such race, gender, sexual orientation, etc. (8)
- ☐ Geographic considerations (3)
- ☐ I was screened out due to my scores (1)
- ☐ IMG status (4)
- ☐ DO school (5)
- ☐ Other (please enter reason) (7) ____
- ☐ I cannot explain (please enter comments/reflections) (6) ____

Display This Question:

If Q9 != 0

Q11 How many of those programs did you interview at?

- ○ 0 (1)
- ○ 1 (2)
- ○ 2 (3)
- ○ 3 (4)

Display This Question:

If Q11 != 0

© 2021 Winkel AF et al. *JAMA Network Open.*

Q12 Of the programs you interviewed at, how many did you rank in your top 3?

◯ 0 (1)

◯ 1 (2)

◯ 2 (3)

◯ 3 (4)

Q13 Overall, how equitable is the CURRENT residency application and selection processes?

◯ Not equitable (1)

◯ Somewhat equitable (2)

◯ Equitable (3)

Display This Question:

If Q13 != Equitable

Q14 Please add comments why it is not equitable and/or describe interventions to make it equitable

____

____

____

____

____

© 2021 Winkel AF et al. *JAMA Network Open.*

Q15 In regards to the number of residency applications an applicant can submit, which of the following is most equitable

- ◯ All applicants are allowed to apply to as many programs as they want (current state) (1)
- ◯ More competitive applicants are limited to total number of applications they can submit (2)
- ◯ All applicants are limited to the same total number of applications they can submit (application cap) (3)
- ◯ Other/none of the above (enter comment) (4) ____

Display This Question:

If Q15 = All applicants are limited to the same total number of applications they can submit (application cap)

*

Q16 How many applications? Please enter a whole number

____

Q17 In regards to the number of interview offers an applicant can accept, which of the following is most equitable

- ◯ All applicants are allowed to interview at as many programs as they want (current state) (1)
- ◯ More competitive applicants are limited to total number of interviews they can accept (2)
- ◯ All applicants are limited to the same total number of interviews they can accept (interview cap) (3)
- ◯ Other/none of the above (enter comment) (4) ____

© 2021 Winkel AF et al. *JAMA Network Open.*

Display This Question:

If Q17 = All applicants are limited to the same total number of interviews they can accept (interview cap)

Q18 How many interviews? Please enter a whole number

____

Q19 Please rank the following resources from most helpful to least helpful during the residency application cycle

______ AAMC Residency Explorer (5)
______ AMA-FREIDA (4)
______ APGO Residency directory (1)
______ Instagram (9)
______ Residency program websites (7)
______ Student Reddit site (6)
______ Twitter (8)
______ Other (10)

Q20 The next two questions are in regards to a hypothetical early result acceptance program (ERAP) similar to Early decision for college as defined below: ● Students would apply to a limited number of programs● Programs would be allowed to fill only half their spots in ERAP● Binding match results announced in September● Applicants who do not secure a position in ERAP, enter the regular application and process

© 2021 Winkel AF et al. *JAMA Network Open.*

Q21 How likely would you have considered participating in ERAP had it been an option?

- ○ Extremely unlikely (16)
- ○ Somewhat unlikely (17)
- ○ Neither likely nor unlikely (18)
- ○ Somewhat likely (19)
- ○ Extremely likely (20)

Q22 If ERAP were an option for applicants, what is the MAXIMUM number of programs an applicant should be limited to when applying for ERAP?

- ○ 1 (1)
- ○ 2 (2)
- ○ 3 (3)
- ○ 4 (4)
- ○ 5 (5)

Q23 Are you couple's matching?

- ○ Yes (1)
- ○ No (2)

© 2021 Winkel AF et al. *JAMA Network Open.*

Q24 Please check your USMLE Step 1 score or COMLEX 1 score

- USMLE (1)
- USMLE 200-220 or COMLEX 488-575 (4)
- USMLE 221-240 or COMLEX 576-660 (5)
- USMLE 241-260 or COMLEX 661-742 (6)
- USMLE >260 or COMLEX >742 (7)

Q25 Please check the status that applies to you when you submitted your application in the 2020-2021 residency application cycle

- US Senior-MD (1)
- US Alumni-MD (7)
- US Senior-DO (4)
- US Alumni-DO (8)
- IMG-US Citizen (5)
- IMG US Citizen Alumni (9)
- IMG-Non US Citizen (6)
- IMG-Non US Citizen Alumni (10)

Q26 How do you self-identify? Please check all that apply

▢ American Indian or Alaska Native (1)

▢ Black or African American (9)

▢ Hispanic, Latino, or of Spanish origin (10)

▢ Native Hawaiian or other Pacific Islander (11)

▢ White (12)

▢ Other (8) ____

Q27 Please share any additional comments/concerns/suggestions about the residency application and interview cycle.

____

____

____

____

____

End of Block: SURVEY INSTRUCTIONS

RRR OBGYN PD Survey 2021

Link to Preview:

https://umich.ca1.qualtrics.com/jfe/preview/SV_6WZSXPezL6RAsbY?Q_CHL=preview&Q_SurveyVersionID=current

Start of Block: SURVEY INSTRUCTIONS

© 2021 Winkel AF et al. *JAMA Network Open.*

Q1 The following questions pertain to the residency application and interview processes in obstetrics and gynecology (OBGYN). Please try to answer all questions to the best of your ability as these are intended to evaluate the changes recommended during the 2020-2021 application cycle. Thank you, we value your input.

Q2 My program followed the following Specialty-Wide Standards for the OBGYN Residency Application and Interview Processes in 2020-2021. CHECK ALL THAT APPLY.

- ▢ November 1, 2020 deadline for final OBGYN application submission (1)
- ▢ December 23, 2020 for final applicant status notification (i.e. waitlist, rejection) (2)
- ▢ November 10 and November 17, 2020 for initial release of interview offers, followed by rolling offers (3)
- ▢ 48 hours minimum after an interview invitation email for the applicant to respond (4)
- ▢ Limited program interview invitations to the number of interview slots available (5)
- ▢ We did not follow any of the above standards (please comment why) (6) ____

© 2021 Winkel AF et al. *JAMA Network Open.*

Q3 Which of the following Specialty-Wide Standards for the OBGYN Residency Application and Interview Processes should be continued in future years? CHECK ALL THAT APPLY

- ☐ Deadline for final OBGYN application submission (1)
- ☐ Deadline for final applicant status notification by programs (i.e. waitlist, rejection) (2)
- ☐ Common interview offer dates, followed by rolling offers (3)
- ☐ 48 hours minimum after an interview invitation email has been sent for the applicant to respond (4)
- ☐ Limiting program interview invitations to the number of interview slots available (5)

Q4 How preferable would it be to have one date for common interview offers instead of two?

- ○ Do not prefer (21)
- ○ Prefer slightly (22)
- ○ Prefer a moderate amount (23)
- ○ Prefer a lot (24)
- ○ Prefer a great deal (25)

© 2021 Winkel AF et al. *JAMA Network Open.*

Q5 What is the earliest that would work for your program for a common date to release interview offers?

- ◯ First Tuesday of October (1)
- ◯ Second Tuesday of October (2)
- ◯ Third Tuesday of October (3)
- ◯ Fourth Tuesday of October (4)
- ◯ First Tuesday of November (5)
- ◯ Other (6) ____

Page Break

Q6 Please check which applicant you would consider for your program. CHECK ALL THAT APPLY.

- ☐ US Senior-MD (1)
- ☐ US Alumni-MD (7)
- ☐ US Senior-DO (4)
- ☐ US Alumni-DO (8)
- ☐ IMG-US Citizen (5)
- ☐ IMG US Citizen Alumni (9)
- ☐ IMG-Non US Citizen (6)
- ☐ IMG-Non US Citizen Alumni (10)

Q7 How many applications did you receive in the 2020-2021 season?

- ○ < 200 (1)
- ○ 201-500 (4)
- ○ 501 – 1000 (5)
- ○ 1001 – 1500 (6)
- ○ >1500 (7)

© 2021 Winkel AF et al. *JAMA Network Open.*

Q8 Please fill in the following as it pertains to the 2020-2021 residency application cycle. Please enter whole numbers- enter 0 if none. If you cannot recall exactly use your best estimate.

◯ Number of categorical positions in your program (7) ____

◯ Number of interviews you offer (8) ____

◯ Number of applicants you interview (9) ____

◯ Number of interview cancellations (10) ____

Q9 Please indicate how virtual interviews affected the number of applicants you interviewed compared to last year

◯ Did not change (1)

◯ Interviewed more applicants than a typical year- Please enter number of additional interviews (2) ____

◯ Interviewed less applicants than a typical year- Please enter how many less interviews (3) ____

Q10 Please rank the following interview options which your program would prefer to offer in the future from most desirable (1) to least desirable (4). Please assume that in person options have no restrictions (as in pre-COVID times).

______ All interviews are conducted exclusively virtually (1)
______ All interviews are conducted virtually with open house in person visit option (2)
______ All interviews are conducted in person (3)
______ Applicants have the option of virtual or in person interview (4)

Q11 How many OBGYN residency spots were unfilled in your program prior to SOAP last year (March 2020)?

- None (1)
- 1 (2)
- 2 (3)
- 3 (4)
- 4 (5)
- 5 or more (6)

Q12 Overall, how equitable is the CURRENT residency application and selection processes?

- Not equitable (1)
- Somewhat equitable (2)
- Equitable (3)

Display This Question:

If Q12 != Equitable

Q13 Please add comments why it is not equitable and/or describe interventions to make it equitable

____

____

____

____

____

© 2021 Winkel AF et al. *JAMA Network Open.*

Q14 In regards to the number of residency applications an applicant can submit, which of the following is most equitable

- ◯ All applicants are allowed to apply to as many programs as they want (current state) (1)
- ◯ More competitive applicants are limited to total number of applications they can submit (2)
- ◯ All applicants are limited to the same total number of applications they can submit (application cap) (3)
- ◯ Other/none of the above (enter comment) (4) ____

Display This Question:

If Q14 = All applicants are limited to the same total number of applications they can submit (application cap)

Q15 How many applications? Please enter a whole number

____

Q16 In regards to the number of interview offers an applicant can accept, which of the following is most equitable

- ◯ All applicants are allowed to interview at as many programs as they want (current state) (1)
- ◯ More competitive applicants are limited to total number of interviews they can accept (2)
- ◯ All applicants are limited to the same total number of interviews they can accept (interview cap) (3)
- ◯ Other/none of the above (enter comment) (4) ____

© 2021 Winkel AF et al. *JAMA Network Open.*

Display This Question:

If Q16 = All applicants are limited to the same total number of interviews they can accept (interview cap)

Q17 How many interviews? Please enter a whole number

____

Q18 The next two questions are in regards to a hypothetical early result acceptance program (ERAP) similar to Early decision for college as defined below: ● Students would apply to a limited number of programs● Programs would be allowed to fill only a portion of their spots in ERAP● Binding match results announced in September● Applicants who do not secure a position in ERAP, enter the regular application and process

Q19 How likely are you to participate in ERAP if it is an option?

- ○ Extremely unlikely (16)
- ○ Somewhat unlikely (17)
- ○ Neither likely nor unlikely (18)
- ○ Somewhat likely (19)
- ○ Extremely likely (20)

© 2021 Winkel AF et al. *JAMA Network Open.*

Q20 If ERAP were an option, what is the MAXIMUM percentage of spots a program should be able to fill in the early match?

- 25% (1)
- 50% (2)
- 75% (3)
- 100% (4)
- Each program can decide (5)

Q21 My program is in CREOG region

- Region 1 (Connecticut, Maine, Massachusetts, Newfoundland, New Hampshire, New York, Nova Scotia, Quebec, Rhode Island, Vermont) (1)
- Region 2 (Delaware, Indiana, Kentucky, Michigan, New Jersey, Ohio, Ontario, Pennsylvania) (2)
- Region 3 (District of Columbia, Florida, Georgia, Maryland, North Carolina, Puerto Rico, South Carolina, Virginia, West Virginia) (3)
- Region 4 (Alabama, Arkansas, Illinois, Iowa, Kansas, Louisiana, Manitoba, Minnesota, Mississippi, Missouri, Nebraska, Oklahoma, Tennessee, Texas, Wisconsin) (4)
- Region 5 (Alberta, Arizona, Armed Forces District, British Columbia, California, Colorado, Hawaii, Nevada, New Mexico, Oregon, Utah, Washington) (5)
- Other (6) ____

© 2021 Winkel AF et al. *JAMA Network Open.*

Q22 My program is

◯ University based (1)

◯ Community based (6)

◯ Both (7)

◯ Other (2) ____

Q23 Please share any additional comments/concerns/suggestions that you may have. You may include comments you heard directly from the applicants.

____

____

____

____

____

End of Block: SURVEY INSTRUCTIONS

RRR OBGYN CD Survey 2021

Preview link

https://umich.ca1.qualtrics.com/jfe/preview/SV_9vpJdkendWTRuSO?Q_CHL=preview&Q_SurveyVersionID=current

---

Start of Block: SURVEY INSTRUCTIONS

© 2021 Winkel AF et al. *JAMA Network Open.*

Q1 The following questions pertain to the residency application and interview processes in obstetrics and gynecology (OBGYN). Please try to answer all questions to the best of your ability as these are intended to evaluate the changes recommended during the 2020-2021 application cycle. Thank you, we value your input.

Q2 Which of the following Specialty-Wide Standards for the OBGYN Residency Application and Interview Processes are you aware of? CHECK ALL THAT APPLY

- ▢ November 1, 2020 as the deadline for final OBGYN application submission (1)
- ▢ December 23, 2020 as the final applicant status notification by programs (i.e. waitlist, rejection) (2)
- ▢ November 10 and November 17, 2020 for initial release of interview offers, followed by rolling offers (3)
- ▢ 48 hours minimum after an interview invitation email has been sent for the applicant to respond (4)
- ▢ Limiting program interview invitations to the number of interview slots available (5)
- ▢ I am not aware of any of the above standards (6)

© 2021 Winkel AF et al. *JAMA Network Open.*

Q3 Rate the importance of each of the following as it pertains to the OBGYN application and interview processes

	Not at all important (1)	Slightly important (2)	Moderately important (3)	Very important (4)	Extremely important (5)
The same application submission dateline for all programs (1)	○	○	○	○	○
A common date for final application status notification to the applicant (i.e. waitlist, rejection) (2)	○	○	○	○	○
Common pre-determined interview offer date(s) for all programs (3)	○	○	○	○	○
A 48-hour period for applicants to respond to an interview offer (4)	○	○	○	○	○
Programs limiting the number of interview offers to slots available (5)	○	○	○	○	○

© 2021 Winkel AF et al. *JAMA Network Open.*

Q4 To how many programs did you recommend students apply to in OBGYN?

- ◯ 1 – 20 (1)
- ◯ 21 – 40 (2)
- ◯ 31 – 50 (3)
- ◯ 51 – 70 (4)
- ◯ > 70 (5)

Q5 Please indicate how virtual interviews affected your recommendations for the number of applications per student?

- ◯ Did not change my recommendation (1)
- ◯ Recommended more applications than a typical year (2)
- ◯ Recommended less applications than a typical year (3)

Q6 How many students at your school applied to OBGYN residency in the 2020-2021 season? (enter a whole number, enter 0 if none)

____

Q7 How many students at your school applying to OBGYN residency did not match in the 2019-2020 season? (enter a whole number, enter 0 if none)

____

© 2021 Winkel AF et al. *JAMA Network Open.*

Q8 Please indicate which students you meet with during these different time points of the residency application cycle.

	All Students (1)	Students I am concerned about (2)	Highly competitive students (5)	Students who request to meet (3)
Prior to application submission (1)	○	○	○	○
After residency interview offers release (2)	○	○	○	○
Prior to rank list submission (3)	○	○	○	○

Q9 Please rank the following resources from most helpful to least helpful during the residency application cycle

______ AAMC Residency Explorer (5)
______ AMA-FREIDA (4)
______ APGO Residency directory (1)
______ Instagram (9)
______ Residency program websites (7)
______ Student Reddit site (6)
______ Twitter (8)
______ Other (10)

© 2021 Winkel AF et al. *JAMA Network Open.*

Q10 Overall, how equitable is the CURRENT residency application and selection processes?

- Not equitable (1)
- Somewhat equitable (2)
- Equitable (3)

Display This Question:

If Q10 != Equitable

Q11 Please add comments why it is not equitable and/or describe interventions to make it equitable

____

____

____

____

____

Q12 In regards to the number of residency applications an applicant can submit, which of the following is most equitable

- All applicants are allowed to apply to as many programs as they want (current state) (1)
- More competitive applicants are limited to total number of applications they can submit (2)
- All applicants are limited to the same total number of applications they can submit (application cap) (3)
- Other/none of the above (enter comment) (4) ____

© 2021 Winkel AF et al. *JAMA Network Open.*

Display This Question:

If Q12 = All applicants are limited to the same total number of applications they can submit (application cap)

Q13 How many applications? Please enter a whole number

____

Q14 In regards to the number of interview offers an applicant can accept, which of the following is most equitable

- ○ All applicants are allowed to interview at as many programs as they want (current state) (1)
- ○ More competitive applicants are limited to total number of interviews they can accept (2)
- ○ All applicants are limited to the same total number of interviews they can accept (interview cap) (3)
- ○ Other/none of the above (enter comment) (4) ____

Display This Question:

If Q14 = All applicants are limited to the same total number of interviews they can accept (interview cap)

Q15 How many interviews? Please enter a whole number

____

Q16 The next two questions are in regards to a hypothetical early result acceptance program (ERAP) similar to Early decision for college as defined below: • Students would apply to a limited number of programs• Programs would be allowed to fill only half their spots in ERAP• Binding match results

© 2021 Winkel AF et al. *JAMA Network Open.*

announced in September● Applicants who do not secure a position in ERAP, enter the regular application and process

Q17 How likely would you have recommended a student participates in ERAP had it been an option?

- Extremely unlikely (16)
- Somewhat unlikely (17)
- Neither likely nor unlikely (18)
- Somewhat likely (19)
- Extremely likely (20)

Q18 If ERAP were an option for applicants, what is the MAXIMUM number of programs an applicant should be limited to when applying for ERAP?

- 1 (1)
- 2 (2)
- 3 (3)
- 4 (4)
- 5 (5)

© 2021 Winkel AF et al. *JAMA Network Open.*

Q19 Which of the following degree(s) does your medical school offer?

○ MD (1)

○ DO (2)

Q20 Please share any additional comments/concerns/suggestions that you may have about the OBGYN Residency Application and Interview Processes. You may include comments you heard directly from the applicants.

____

____

____

____

____

End of Block: SURVEY INSTRUCTIONS

RRR OBGYN GSA Survey 2021

Preview link:

https://umich.ca1.qualtrics.com/jfe/preview/SV_0GOkKS1FrCBAgJM?Q_CHL=preview&Q_SurveyVersionID=current

---

Start of Block: SURVEY INSTRUCTIONS

Q1 The following questions pertain to the residency application and interview processes in obstetrics and gynecology (OBGYN). Please try to answer all questions to the best of your ability as these are intended to evaluate the changes recommended during the 2020-2021 application cycle. Thank you, we value your input.

© 2021 Winkel AF et al. *JAMA Network Open.*

Q2 Which of the following Specialty-Wide Standards for the OBGYN Residency Application and Interview Processes are you aware of? CHECK ALL THAT APPLY

- ▢ November 1, 2020 as the deadline for final OBGYN application submission (1)
- ▢ December 23, 2020 as the final applicant status notification by programs (i.e. waitlist, rejection) (2)
- ▢ November 10 and November 17, 2020 for initial release of interview offers, followed by rolling offers (3)
- ▢ 48 hours minimum after an interview invitation email has been sent for the applicant to respond (4)
- ▢ Limiting program interview invitations to the number of interview slots available (5)
- ▢ I am not aware of any of the above standards (6)

© 2021 Winkel AF et al. *JAMA Network Open.*

Q3 Rate the importance of each of the following as it pertains to the OBGYN application and interview processes

	Not at all important (1)	Slightly important (2)	Moderately important (3)	Very important (4)	Extremely important (5)
The same application submission dateline for all programs (1)	○	○	○	○	○
A common date for final application status notification to the applicant (i.e. waitlist, rejection) (2)	○	○	○	○	○
Common pre-determined interview offer date(s) for all programs (3)	○	○	○	○	○
A 48-hour period for applicants to respond to an interview offer (4)	○	○	○	○	○
Programs limiting the number of interview offers to slots available (5)	○	○	○	○	○

© 2021 Winkel AF et al. *JAMA Network Open.*

Q4 To how many programs did you recommend students apply to in OBGYN?

- ◯ 1 – 20 (1)
- ◯ 21 – 40 (2)
- ◯ 31 – 50 (3)
- ◯ 51 – 70 (4)
- ◯ > 70 (5)
- ◯ Not applicable (6)

Q5 Please indicate how virtual interviews affected your recommendations for the number of applications per student?

- ◯ Did not change my recommendation (1)
- ◯ Recommended more applications than a typical year (2)
- ◯ Recommended less applications than a typical year (3)

Q6 How many students at your school applied to OBGYN residency in the 2020-2021 season? (enter a whole number, enter 0 if none)

____

© 2021 Winkel AF et al. *JAMA Network Open.*

Q7 How many students at your school applying to OBGYN residency did not match in the last cycle, 2019-2020 season? (enter a whole number, enter 0 if none)

____

Q8 Please rank the following resources from most helpful to least helpful during the residency application cycle

______ AAMC Residency Explorer (5)
______ AMA-FREIDA (4)
______ APGO Residency directory (1)
______ Instagram (9)
______ Residency program websites (7)
______ Student Reddit site (6)
______ Twitter (8)
______ Other (10)

Q9 Overall, how equitable is the CURRENT residency application and selection processes?

- Not equitable (1)
- Somewhat equitable (2)
- Equitable (3)

Display This Question:

If Q9 != Equitable

Q10 Please add comments why it is not equitable and/or describe interventions to make it equitable

____

____

____

© 2021 Winkel AF et al. *JAMA Network Open.*

____

____

Q11 In regards to the number of residency applications an applicant can submit, which of the following is most equitable

- ◯ All applicants are allowed to apply to as many programs as they want (current state) (1)
- ◯ More competitive applicants are limited to total number of applications they can submit (2)
- ◯ All applicants are limited to the same total number of applications they can submit (application cap) (3)
- ◯ Other/none of the above (enter comment) (4) ____

Display This Question:

If Q11 = All applicants are limited to the same total number of applications they can submit (application cap)

*

Q12 How many applications? Please enter a whole number

____

© 2021 Winkel AF et al. *JAMA Network Open.*

Q13 In regards to the number of interview offers an applicant can accept, which of the following is most equitable

◯ All applicants are allowed to interview at as many programs as they want (current state) (1)

◯ More competitive applicants are limited to total number of interviews they can accept (2)

◯ All applicants are limited to the same total number of interviews they can accept (interview cap) (3)

◯ Other/none of the above (enter comment) (4) ____

Display This Question:

If Q13 = All applicants are limited to the same total number of interviews they can accept (interview cap)

*

Q14 How many interviews? Please enter a whole number

____

Q15 The next two questions are in regards to a hypothetical early result acceptance program (ERAP) similar to Early decision for college as defined below: ● Students would apply to a limited number of programs● Programs would be allowed to fill only half their spots in ERAP● Binding match results announced in September● Applicants who do not secure a position in ERAP, enter the regular application and process

---

© 2021 Winkel AF et al. *JAMA Network Open.*

Q16 How likely would you have recommended a student participates in ERAP had it been an option?

- Extremely unlikely (16)
- Somewhat unlikely (17)
- Neither likely nor unlikely (18)
- Somewhat likely (19)
- Extremely likely (20)

Q17 If ERAP were an option for applicants, what is the MAXIMUM number of programs an applicant should be limited to when applying for ERAP?

- 1 (1)
- 2 (2)
- 3 (3)
- 4 (4)
- 5 (5)

Q18 Which of the following degree(s) does your medical school offer?

- MD (1)
- DO (2)

© 2021 Winkel AF et al. *JAMA Network Open.*

Q19 Please share any additional comments/concerns/suggestions that you may have about OBGYN Residency Application and Interview Processes.   You may include comments you heard directly from the applicants.

____

____

____

____

____

End of Block: SURVEY INSTRUCTIONS

© 2021 Winkel AF et al. *JAMA Network Open.*
